# Population-based changes in oral health–related quality of life in Australia

**DOI:** 10.1186/s12955-026-02570-4

**Published:** 2026-06-19

**Authors:** Xiangqun Ju, Liana Luzzi, Lisa M. Jamieson

**Affiliations:** Australian Research Centre for Population Oral Health, Adelaide Dental School, Adelaide University, Level 4, 50 Rundle Mall Plaza, Adelaide, South Australia 5005 Australia

**Keywords:** Oral health-related quality of life (OHRQoL), The oral health impact profile (OHIP-14), General health, Oral health, Tooth loss

## Abstract

**Background:**

Good oral health is crucial for overall well-being; yet many people experience pain, discomfort, or social difficulties due to oral problems. Understanding how Australians’ oral health and quality of life have changed over time helps identify whether national dental policies and access to care have improved. The study aimed to assess temporal changes in oral health-related quality of life (OHRQoL) and identify associated risk indicators using the short Oral Health Impact Profile (OHIP-14) instrument among Australian adults.

**Methods:**

Data were sourced from the two most recent nationally representative surveys in Australia: National Survey of Adult Oral Health, NSAOH-1 (2004–06) and NSAOH-2 (2017–18). Both are population-based cross-sectional studies conducted in Australia, targeting adults aged 15 years and older. The outcome of interest was oral health–related quality of life (OHRQoL), measured using the validated short-form Oral Health Impact Profile (OHIP-14). Survey-weighted negative binomial regression models were employed to estimate both unadjusted and adjusted mean ratios (MRs) and their 95% confidence intervals (CIs) for OHIP-14 scores. Blinder–Oaxaca decomposition analysis was used to assess the contribution of changes in measured population characteristics to differences in mean OHIP-14 scores between two surveys.

**Results:**

In total, 4,170 participants completed the OHIP-14 questionnaire in 2004–06, and 2,836 did so in 2017–18. Between the two survey waves, the mean OHIP-14 score decreased from 7.3 to 5.5. After adjusting for all covariates, participants in NSAOH-1 had a 61% higher mean OHIP-14 score compared to those in NSAOH-2 (MR = 1.61; 95% CI: 1.53–1.70). Higher OHIP-14 scores were found among older adults, lower-educated, lower-income individuals, those with poorer health, smokers, frequent toothache sufferers, and people whose last dental visit was for a problem. The decomposition analysis showed that 41% of the difference in mean OHIP-14 scores was explained by measured population characteristics, Household income, and usual reason for dental visiting made the largest contributions to the explained component (80%), followed by smoking status (15%) and education level (9%).

**Conclusion:**

OHRQoL among Australian adults improved over 14 years, reflected by lower mean OHIP-14 scores in NSAOH-2 than NSAOH-1. However, poorer OHRQoL remained associated with older age, female sex, socioeconomic disadvantage, poorer self-rated health and oral health, smoking, toothache, and problem-oriented dental visiting. Decomposition analysis suggested that changes in income and dental visiting patterns partly explained this improvement.

## Background

Oral health-related quality of life (OHRQoL) is a multidimensional construct that reflects the impact of oral health on an individual’s overall well-being, including functional, psychological, and social aspects. It goes beyond clinical indicators to capture how oral conditions affect daily life, such as eating, speaking, self-esteem, and social interactions. A shortened version of the Oral Health Impact Profile (OHIP-14) is a widely validated instrument commonly used to assess these perceived impacts at both individual and population levels [1]. Population-based studies from several countries have reported relatively low mean OHIP-14 scores, commonly ranging from approximately 4.0 to 6.5 on the standard 0–56 scale. For example, mean scores of 4.1 among Norwegian adults, 5.7 among adults in England, and 6.3 among Swedish adults have been reported, indicating generally modest average functional, psychological, and social impacts of oral health in general adult populations [2]. The 2004-06 National Oral Health Survey in Australia reported a mean OHIP-14 score of 7.2 among adults [3]. In comparison, regional Aboriginal adults in South Australia had a substantially higher mean score of 21.4 [4], exceeding the average scores reported in many international general adult populations.

Although Australian oral health trends have been widely reported, less is known about how perceived oral health outcomes have changed over time at the population level, and whether these changes reflect shifts in population composition or changing social and behavioural gradients in OHRQoL. This distinction is important because Australia has experienced demographic ageing, persistent socioeconomic inequalities, and changes in dental service use and access over recent decades, including cost-related avoidance of dental care [5, 6]. These broader population and health system changes may influence not only the prevalence of oral health impacts, but also the extent to which risk indicators such as age, income, education, smoking, dental visiting patterns, and residential location are associated with poorer OHRQoL.

Dental diseases, highly prevalent and particularly periodontal disease, are major contributors to diminished oral health-related quality of life (OHRQoL) due to their combined physical and psychosocial impacts [7, 8]. Tooth loss, often the result of such conditions, can lead to functional limitations such as difficulty eating and speaking, as well as aesthetic concerns that may cause embarrassment. These consequences can impair masticatory efficiency and alter dietary habits, ultimately reducing nutrient intake and further compromising individuals’ OHRQoL [9, 10]. Previous studies have also identified systemic conditions, behavioural factors, and socioeconomic disadvantage as important risk indicators for poorer OHRQoL [11–16]. However, it remains unclear whether changes in OHRQoL among Australian adults between national survey waves are primarily explained by changes in the distribution of these population characteristics or by changes in their associations with OHRQoL.

Therefore, this study aimed to examine temporal changes in OHRQoL among Australian adults between the 2004–06 and 2017–18 National Surveys of Adult Oral Health using the OHIP-14 instrument. We further aimed to identify key sociodemographic, behavioural, and oral health-related risk indicators associated with OHRQoL, and to determine whether changes in mean OHIP-14 scores between survey waves were attributable to changes in population composition, changes in the associations between population characteristics and OHRQoL, or both.

## Methods

Strengthening the Reporting of Observational Studies in Epidemiology (STROBE) guidelines were used for transparent and comprehensive reporting.

### Study design, sample size and data collection

Data for this analysis were drawn from the most recent comparable national sources: the National Survey of Adult Oral Health (NSAOH) conducted in 2004–06 (NSAOH-1) and 2017–18 (NSAOH-2). Both surveys were population-based cross-sectional studies of Australian adults aged 15 years and older. Participants were recruited from metropolitan and regional areas across all states and territories using a three-stage stratified sampling design, with individuals randomly selected from sampled households. Detailed methodology is available in previously published reports [17, 18]. Data were weighted following standard procedures for clustered samples. Selected individuals were invited to complete a questionnaire to self-report their oral health and related characteristics. Data were collected via computer-assisted telephone interviews (CATI) in 2004–06, and through either CATI or an online questionnaire in 2017–18. The response rates were 49% for NSAOH-1 and 40% for NSAOH-2.

### Ethics approval and consent to participate

This study is reported in accordance with the Declaration of Helsinki. Both NSAOH-1 (H-001-2004) and NSAOH-2 (H-2016-182) were reviewed and approved by the University of Adelaide Human Research Ethics Committee. All participants provided written informed consent, and parents of participants under the age of 16 provided signed informed consent when participants under the age of 16 were recruited.

### Variables

#### Outcome variable

The outcome variable was oral health–related quality of life (OHRQoL), assessed using the validated short-form Oral Health Impact Profile (OHIP-14) instrument (1). The OHIP-14 captures seven conceptual dimensions of oral health that reflect its impact on broader aspects of daily life: functional limitation, physical pain, psychological discomfort, physical disability, psychological disability, social disability, and handicap [19]. Responses were recorded on a five-point Likert scale: 0 = Never, 1 = Hardly ever, 2 = Occasionally, 3 = Fairly often, and 4 = Very often. Severity scores were calculated by summing all item responses, yielding a total score ranging from 0 to 56. Higher scores indicate a greater negative impact on individuals’ self-perceived oral health–related quality of life (OHRQoL).

#### Exposure variable

The exposure variable was the survey time point, defined by two waves: NSAOH-1 and NSAOH-2.

#### Covariates

Covariates consisted of socio-demographic factors, including age (15–34, 35–54, 55–74, 75 + years), sex (Male vs. Female), residential location (Major city vs. Regional/remote), education level (< High school, High school, Trade to diploma, ≥ University), income (<$30K, ≥$30 to <$60K, ≥$60 to <$100K, ≥$100K), employment status (Unemployed vs. Employed) and possession of a means-tested health care card (Yes vs. No); general health indicators included self-rated general health (Fair/poor, Good, Excellent/Very good), presence of diabetes (Had vs. Not), and tobacco use (Currently, Used smoke, Never); oral health status included self-reported number of teeth, self-rated oral health (Fair/poor, Good, Excellent/Very good) and history of toothache (Very often/often, Sometimes, Hardly/never); and oral health-related behaviours included timing of the last dental visit (One year or more vs. Less than one year), usual reason for visiting the dentist (Problem vs. Check-up), and frequency of regular dental visit (> one year vs. ≤ One year).

### Statistical analysis

Data management and computation of summary variables were performed using SAS software (version 9.4; SAS Institute Inc., Cary, NC, USA) and Stata 14. Survey weights were applied to account for the complex sampling design.

The analysis first involved calculating univariate statistics to describe the distribution of sample characteristics and the corresponding mean OHIP-14 scores with 95% confidence intervals (CIs). Non-overlapping 95% CIs were interpreted as a conservative indicator of statistical significance, while differences assessed using the Mann–Whitney U test were considered significant at *p* < 0.05. Subsequently, survey-weighted negative binomial regression models were employed. Crude and adjusted mean ratios (MRs) with their 95% confidence intervals (CIs) were calculated for the mean OHIP-14 scores. The exposure variable and covariates were entered into multivariable models in four sequential steps: Model 1: unadjusted (crude) model, Model 2: survey-adjusted and controlled for socio-demographic factors, Model 3: Model 2 additionally adjusted for general health indicators, Model 4 (Full model): Model 3 further adjusted for oral health status and related behaviours. We further conducted Oaxaca-Blinder decomposition analysis to examine whether the observed change in OHIP 14 scores between survey waves was attributable to changes in population composition, changes in the associations between population characteristics and OHRQoL, or both. It is a counterfactual analysis that enables investigation into what actually happened and what would have happened in the absence of the intervention [20].

## Results

A total of 4,170 participants completed the OHIP-14 questionnaire in 2004–06, and 2,836 participants did so in 2017–18.

Table 1 summarises the sample characteristics and mean OHIP-14 scores across the two survey periods. Overall, the 2017–18 sample appeared more socioeconomically advantaged than the 2004–06 sample, with higher proportions of participants living in Major Cities, reporting trade or diploma-level education, having a household income of ≥$100,000, and attending dental check-ups. In contrast, the 2004–06 sample had higher proportions of participants with lower educational attainment, lower-to-middle household income, current smoking, and problem-oriented dental visiting. However, despite these favourable socioeconomic and dental attendance patterns in 2017–18, the latter survey also included higher proportions of participants reporting fair/poor general health, fair/poor oral health, and more frequent toothache. These contrasting compositional shifts suggest that temporal changes in mean OHIP-14 scores should be interpreted in relation to both improvements in some social and service-use indicators and worsening self-reported health and oral health indicators.

**Table 1:** Sample characteristics and mean scores of OHIP14 (weighted)

	NSAOH 2004-06			NSAOH 2017-18		
	Number	% (95% CI)	Mean (95% CI)	Number	% (95% CI)	Mean (95% CI)
**Total**	4170	100	7.3 (7.1-7.5)	2836	100	5.5 (5.2-5.7)
**Socio-demographic characteristics**						
**Age groups (years**)						
15-34	754	37.2 (34.1-40.3)	6.9 (6.3-7.4)	558	36.0 (32.2-39.7)	4.6 (4.1-5.0)
35-54	1674	37.3 (34.9-39.6)	8.0 (7.6-8.4)	814	33.6 (30.4-36.7)	5.9 (5.3-6.4)
55-74	1494	20.3 (18.6-22.0)	7.3 (6.9-7.7)	1149	23.7 (21.3-26.2)	5.9 (5.4-6.4)
≥ 75	248	5.2 (4.3-6.2)	5.7 (4.8-6.5)	315	6.7 (5.5-7.9)	6.5 (5.4-7.7)
**Sex**						
Male	1604	50.0 (47.4-52.6)	6.8 (6.5-7.2)	1218	49.6 (46.1-53.0)	5.2 (4.8-5.6)
Female	2566	50.0 (47.4-52.6)	7.8 (7.4-8.1)	1618	50.4 (47.0-53.9)	5.7 (5.3-6.1)
**Location**						
Regional/remote	1522	34.9 (32.8-36.9)	7.6 (7.2-8.0)	1212	27.2 (23.2-31.1)	5.7 (5.3-6.1)
Major city	2648	65.1 (63.1-67.2)	6.8 (6.8-7.4)	1624	72.8 (68.9-76.8)	5.4 (5.0-5.7)
**Education level**						
< High school	1103	24.3 (22.1-26.5)	8.4 (7.9-9.0)	377	13.1 (10.6-15.6)	6.5 (5.6-7.5)
High school	517	15.4 (13.2-17.6)	7.2 (6.5-7.9)	158	8.9 (6.5-11.4)	5.7 (4.6-6.8)
Trade to diploma	1169	27.5 (25.0-29.9)	8.0 (7.5-8.5)	1184	48.3 (44.5-52.1)	6.2 (5.7-6.7)
≥University	1372	32.9 (30.1-35.7)	5.9 (5.6-6.3)	1114	29.6 (26.0-33.2)	3.7 (3.4-4.0)
**Income**						
<$30K	1253	24.1 (21.7-26.6)	9.9 (9.3-10.4)	638	22.6 (19.2-26.0)	7.4 (6.7-8.2)
≥$30 to <$60K	1219	29.4 (26.9-31.9)	7.8 (7.3-8.3)	541	22.3 (19.1-25.6)	7.5 (6.7-8.3)
≥$60 to <$100K	970	30.5 (27.8-33.2)	5.8 (5.4-6.1)	499	21.2 (18.3-24.1)	4.7 (4.1-5.2)
≥$100K	471	16.0 (13.8-18.2)	5.8 (5.2-6.4)	733	33.9 (30.0-37.7)	3.5 (3.1-3.8)
**Employ status**						
Unemployed	1726	35.2 (32.6-37.8)	8.2 (7.8-8.7)	1285	39.9 (36.2-43.6)	6.9 (6.3-7.4)
Employed	2349	64.8 (62.2-67.4)	6.9 (6.6 -7.2)	1539	60.1 (56.4-63.8)	4.5 (4.2-4.8)
**Card holder**						
Yes	1267	25.5 (23.1-28.0)	9.8 (9.2-10.3)	1021	30.0 (26.5-33.6)	7.9 (7.3-8.5)
No	2898	74.5 (72.0-76.9)	6.5 (6.2-6.7)	1807	70.0 (66.4-73.5)	4.4 (4.1-4.7)
**General health**						
**Self-rated general health**						
Fair/Poor	456	9.1 (7.8-10.4)	12.5 (11.5-13.6)	418	14.2 (11.8-16.6)	10.5 (9.5-11.5)
Good	1192	27.8 (25.3-30.3)	8.4 (8.0-8.9)	990	35.1 (31.7-38.5)	6.2 (5.7-6.6)
Excel/Very good	2520	63.1 (60.4-65.8)	6.0 (5.8-6.3)	1421	50.7 (47.0-54.4)	3.6 (3.3-3.9)
**Diabetes**						
Had	213	4.3 (3.2-5.5)	9.4 (8.1-10.8)	246	5.7 (4.4-7.0)	6.0 (4.7-7.2)
Not had	3956	95.7 (94.5-96.8)	7.2 (7.0-7.4)	2573	94.3 (93.0-95.6)	5.4 (5.2-5.7)
**Tobacco smoke status**						
Currently	578	15.1 (13.2-17.1)	9.3 (8.5-10.0)	185	8.0 (5.9-10.1)	9.3 (7.6-11.0)
Used smoke	1315	27.3 (25.1-29.5)	7.7 (7.3-8.1)	954	28.7 (25.6-31.8)	7.1 (6.6-7.7)
Never	2277	57.5 (55.0-60.1)	6.6 (6.3-6.9)	1681	63.3 (59.9-66.6)	4.2 (3.9-4.5)
**Oral health**						
**Self-reported Number of teeth**						
<21	604	9.2 (8.1-10.3)	10.5 (9.7-11.2)	391	8.8 (7.3-10.3)	10.8 (9.6-11.9)
≥ 21	3566	90.8 (89.7-91.9)	7.0 (6.7-7.2)	2440	91.2 (89.7-92.7)	4.9 (4.7-5.2)
**Self-rated oral health**						
Fair/Poor	716	16.0 (13.9-18.0)	14.6 (13.9-15.4)	656	23.5 (20.1-27.0)	11.5 (10.6-12.3)
Good	1630	39.2 (36.3-42.0)	7.5 (7.1-7.8)	1046	36.2 (32.7-39.7)	4.9 (4.5-5.3)
Excel/V good	1822	44.9 (42.3-47.5)	4.5 (4.3-4.8)	1119	40.3 (36.6-43.9)	2.4 (2.2-2.6)
**Experience of a toothache**						
Very often/Often	213	5.3 (4.1-6.4)	15.6 (14.1-17.1)	115	4.8 (3.3-6.4)	17.1 (15.2-19.1)
Sometimes	379	9.3 (7.4-11.1)	14.0 (12.9-15.1)	368	17.8 (15.2-20.3)	9.1 (8.1-10.1)
Hardly/Never	3575	85.5 (83.3-87.6)	6.1 (5.9-6.3)	2351	77.4 (74.5-80.3)	3.9 (3.7-4.1)
**Oral health-related behaviours**						
**Last dental visit**						
One year or more	1537	40.3 (37.6-42.9)	7.4 (7.0-7.8)	1169	45.2 (41.3-49.1)	6.0 (5.5-6.4)
Less than one year	2630	59.7 (57.1-62.4)	7.2 (7.0-7.5)	1664	54.8 (50.9-58.7)	4.9 (4.6-5.3)
**Usual reason for a dental visit**						
Problem	1780	41.5 (38.6-44.3)	9.6 (9.2-10.1)	950	33.7 (30.2-37.1)	8.6 (7.9-9.2)
Check-up	2375	58.5 (55.7-61.4)	5.7 (5.4-5.9)	1850	66.3 (62.9-69.8)	3.8 (3.5-4.0)
**Regular dental visit**						
> One year	1895	46.8 (43.9-49.6)	7.5 (7.2-7.9)	1183	45.1 (41.1-49.2)	6.3 (5.8-6.8)
≤ One year	2259	53.2 (50.4-56.1)	7.1 (6.8-7.4)	1603	54.9 (50.8-58.9)	4.7 (4.4-5.0)

**Fig. 1:**
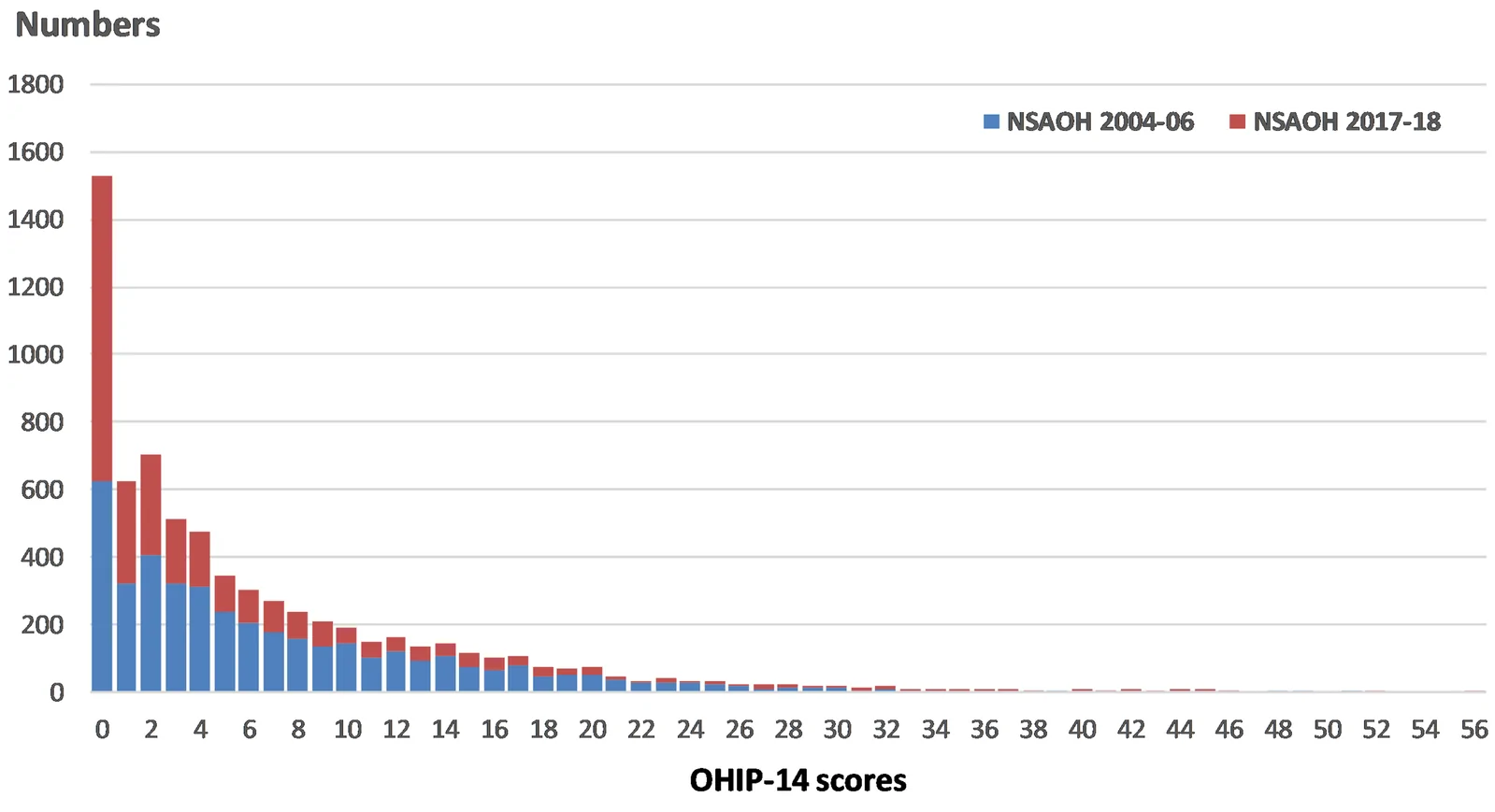
The distribution of mean OHIP-14 scores in both NSAOH 2004-06 and NSAOH 2017-18. Caption: The histogram shows positively skewed OHIP-14 scores, with most reporting minimal impacts and a smaller subgroup experiencing substantially poorer outcomes

Between the two survey periods, the mean OHIP-14 score declined substantially from 7.3 in 2004–06 to 5.5 in 2017–18 (Table 1; Fig. 1), indicating an overall improvement in OHRQoL. This improvement was observed across a wide range of sociodemographic, health, behavioural, and dental service-use groups, suggesting that reductions in perceived oral health impacts were not confined to a single population subgroup. Declines were evident across age, sex, residential location, most education and income groups, employment status, health card status, diabetes status, smoking status, number of teeth, self-rated oral health, toothache experience, and dental visiting patterns. Notably, improvements were also observed among participants reporting fair/poor self-rated oral health, whose mean OHIP-14 score declined from 14.6 to 11.5, although this group continued to experience a substantially higher burden of oral health impacts than those reporting better oral health. Overall, these findings suggest broad-based improvements in OHRQoL over time, while also indicating that important inequalities in perceived oral health impacts persisted across population subgroups.

**Fig. 2:**
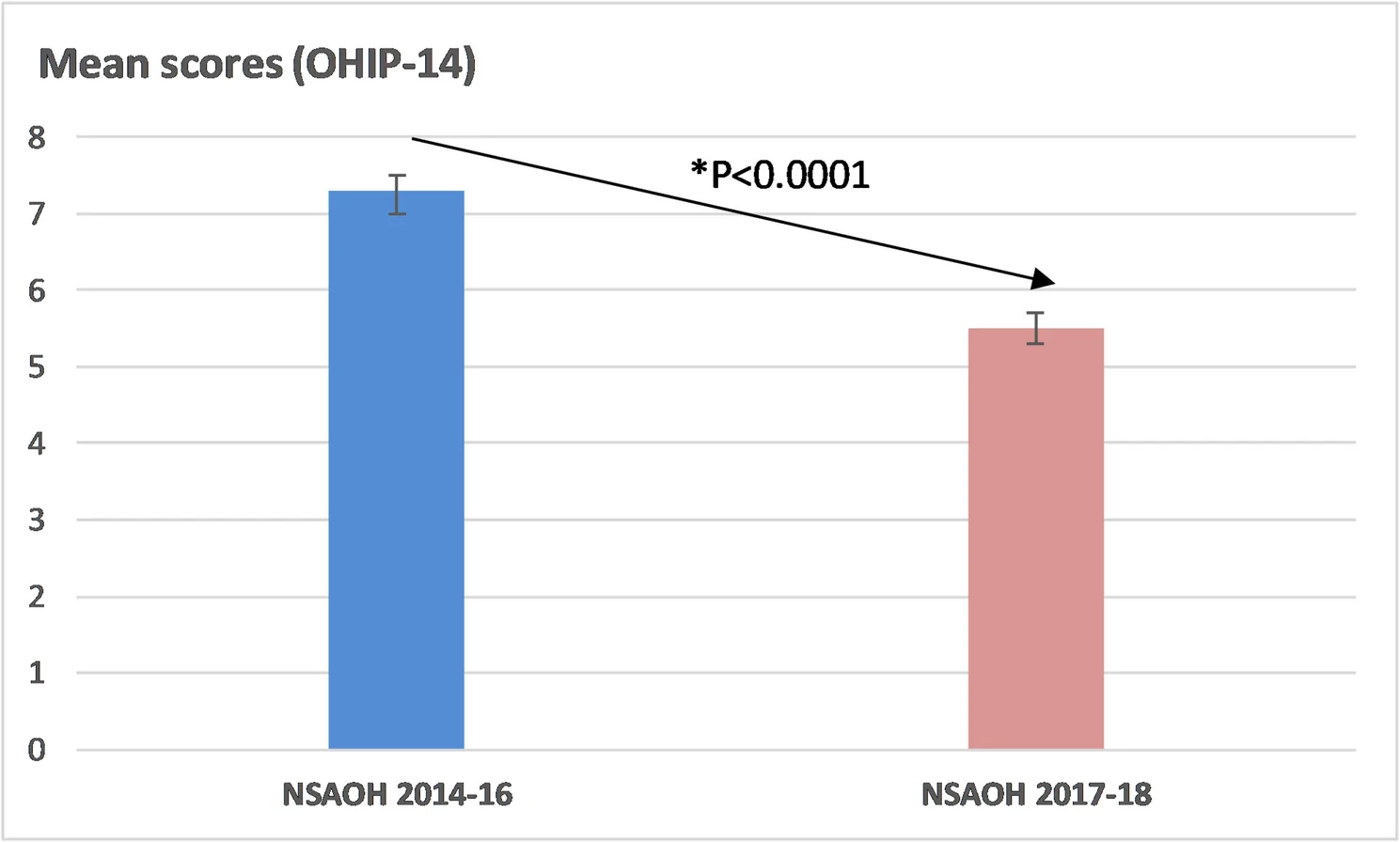
Mean OHIP-14 scores across surveys. Caption: This bar chart shows that the mean OHIP-14 score declined substantially from NSAOH 2004-06 to NSAOH 2017-18

Figure 1 presents the distribution of mean OHIP-14 scores in both NSAOH-1 and NSAOH-2. In both samples, the scores were positively skewed, indicating that most participants reported minimal impacts on oral health-related quality of life, while a smaller subgroup experienced substantially poorer outcomes. The mean OHIP-14 scores were 7.3 in NSAOH-1 and 5.5 in NSAOH-2, with the difference being statistically significant (*p* < 0.0001) (Fig. 2).

Table 2 presents the multivariable analyses comparing the two survey waves and assessing the association between the survey time point and the mean OHIP-14 score among Australian adults. In unadjusted analyses, participants in NSAOH-1 had an 11% higher mean OHIP-14 score compared to those in NSAOH-2 (MR = 1.37; 95% CI: 1.31–1.44). After adjusting for all covariates, the mean OHIP-14 score remained 61% higher in NSAOH-1 participants compared to NSAOH-2 (MR = 1.61; 95% CI: 1.53–1.70). Higher mean OHIP-14 scores were also associated with older age, females, lower educational attainment, lower household income, poorer self-rated general and oral health, current or former smoking, more frequent toothache, and problem-oriented dental visiting. Overall, these findings suggest that poorer OHRQoL in the earlier survey persisted after accounting for differences in population composition and key risk indicators.

**Table 2:** Multivariable association between OHIP-14 and surveys and covariates (MR, 95% CI) (Weighted)

	Model 1	Model 2	Model 3	Model 4
	MR (95% CI)	MR (95% CI)	MR (95% CI)	MR (95% CI)
**Surveys**				
NSAOH 1	1.37 (1.31-1.44)	1.34 (1.27-1.41)	1.45 (1.37-1.53)	1.61 (1.53-1.70)
NSAOH 2	ref	ref	ref	ref
**Age groups (years**)				
≥ 75	1.21 (1.14-1.29)	1.17 (1.10-1.25)	1.10 (1.03-1.17)	1.09 (1.03-1.16)
55-74	1.13 (1.05-1.21)	0.89 (0.83-0.96)	0.85 (0.79-0.92)	0.87 (0.81-0.94)
35-54	1.04 (0.93-1.16)	0.64 (0.57-0.73)	0.63 (0.56-0.71)	0.66 (0.58-0.74)
15-34	ref	ref	ref	ref
**Sex**				
Female	1.09 (1.03-1.14)	1.04 (0.99-1.10)	1.09 (1.04-1.15)	1.06 (1.11-1.08)
Male	ref	ref	ref	
**Location**				
Regional/remote	1.10 (1.04-1.16)	0.99 (0.93-1.04)	0.98 (0.93-1.03)	0.97 (0.93-1.02)
Major city	ref	ref	ref	
**Education level**				
< High school	1.54 (1.44-1.66)	1.32 (1.22-1.44)	1.21 (1.12-1.32)	1.14 (1.06-1.23)
High school	1.31 (1.20-1.42)	1.14 (1.04-1.25)	1.08 (0.98-1.18)	0.98 (0.90-1.06)
Trade to diploma	1.35 (1.27-1.43)	1.29 (1.21-1.38)	1.17 (1.10-1.25)	1.12 (1.05-1.19)
≥University	ref	ref	ref	ref
**Income**				
<$30K	2.19 (2.02-2.36)	1.77 (1.60-1.96)	1.60 (1.44-1.77)	1.34 (1.22-1.47)
≥$30 to <$60K	1.82 (1.68-1.96)	1.58 (1.46-1.72)	1.49 (1.38-1.62)	1.25 (1.16-1.35)
≥$60 to <$100K	1.34 (1.24-1.45)	1.22 (1.13-1.32)	1.16 (1.07-1.25)	1.12 (1.05-1.21)
≥$100K	ref	ref	ref	ref
**Employ status**				
Unemployed	1.29 (1.23-1.36)	1.04 (0.97-1.11)	0.98 (0.92-1.05)	0.96 (0.90-1.02)
Employed	ref	ref	ref	ref
**Card holder**				
Yes	1.57 (1.43-1.65)	1.27 (1.17-1.37)	1.21 (1.12-1.31)	1.08 (1.00-1.15)
No	ref	ref	ref	ref
**Self-rated general health**				
Fair/Poor	2.19 (2.04-2.36)		2.03 (1.87-2.20)	1.22 (1.13-1.32)
Good	1.41 (1.33-1.49)		1.42 (1.34-1.51)	1.07 (1.01-1.13)
Excel/Very good	ref		ref	ref
**Diabetes**				
Had	1.21 (1.09-1.35)		1.02 (0.91-1.13)	0.97 (0.87-1.07)
Not had	ref		ref	ref
**Tobacco smoke status**				
Currently	1.76 (1.63-1.90)		1.38 (1.27-1.49)	1.12 (1.04-1.20)
Used smoke	1.33 (1.26-1.41)		1.31 (1.23-1.39)	1.17 (1.11-1.24)
Never	ref		ref	ref
**Self-reported number of teeth**				
<21	1.76 (1.63-1.90)			1.43 (1.32-1.54)
≥ 21	ref			ref
**Self-rated oral health**				
Fair/Poor	3.55 (3.34-3.77)			2.52 (2.34-2.71)
Good	1.80 (1.71-1.90)			1.60 (1.51-1.70)
Excel/V good	ref			ref
**Experience of a toothache**				
Very often/Often	3.15 (2.85-3.48)			2.15 (1.95-2.37)
Sometimes	2.09 (1.95-2.23)			1.74 (1.63-1.87)
Hardly/Never	ref			ref
**Last dental visit**				
One year or more	1.08 (1.03-1.14)			0.95 (0.90-1.01)
Less than one year	ref			ref
**Usual reason for a dental visit**				
Problem	1.95 (1.86-2.05)			1.23 (1.17-1.31)
Check-up	ref			ref
**Regular dental visit**				
> One year	1.21 (1.15-1.128)			0.91 (0.85-1.02)
≤ One year	ref			ref

**Notes**: MR: Mean ratio; Model 1: Unadjusted (crude) model, Model 2: Survey-adjusted and controlled for socio-demographic factors, Model 3: Model 2 additionally adjusted for general health indicators, Model 4 (Full model): Model 3 further adjusted for oral health status and related behaviours

The decomposition analysis (Table 3) showed that 41% of the difference in mean OHIP-14 scores between NSAOH-1 and NSAOH-2 was explained by changes in the included population characteristics, while 59% remained unexplained. Among the explained components, household income and usual reason for dental visit made the largest contributions, accounting for 40.8% and 41.5%, respectively. Tobacco smoking status and education level also contributed to the observed difference, explaining 15.0% and 9.0%, respectively. These findings suggest that changes in socioeconomic composition and dental visiting patterns were the main measured factors contributing to the decline in mean OHIP-14 scores between survey waves.

**Table 3:** Decomposition of changes in mean OHIP-14 scores among Australian adults aged 15+ years between NSAOH 1 (2004–06) and NSAOH 2 (2017–18)

	NSAOH			
Mean OHIP14(NSAOH 1)	7.377			
Mean OHIP14 (NSAOH 2)	5.473			
Raw differential	1.904			
Due to endowments (E)	0.784			
Due to coefficients (C)	1.132			
Due to interaction (CE)	-0.003			
Explained %	41.0			
Unexplained %	59.0			
**Explanatory variables**	**E**	**C**	**CE**	**Proportion explained (%)**
Age group	0.040	0.999	-0.029	3.15
Sex	-0.007	0.484	0.011	-0.10
Location	-0.015	0.019	0.003	-1.81
Education level	0.099	0.803	-0.054	**9.03** ^*^
Income	0.175	-1.928	0.261	**40.77** ^***^
Employ status	0.001	-0.269	0.007	0.59
Diabetes	0.021	0.173	-0.031	0.90
Tobacco smoke status	0.216	-1.614	-0.153	**15.44** ^**^
Self-reported Number of teeth	0.023	-0.072	-0.006	2.66
Experience of a toothache	-0.028	-1.043	0.005	-3.36
Usual reason for a dental visit	0.290	0.169	0.065	**41.49** ^***^
Regular dental visit	-0.029	-0.510	-0.081	-8.78
**Total**	0.784	1.132	-0.003	100

Notes: ***p-value<0.0001; **p-value<0.01; *p-value<0.05

Coefficients were estimated from the pooled regression model. The decomposition separates the overall difference in outcomes into three components: differences in endowments or observed characteristics (E), differences in coefficients or associations between characteristics and the outcome (C), and the interaction between endowments and coefficients (CE). The explained component represents the proportion of the outcome difference attributable to differences in the distribution of observed explanatory variables between groups. The unexplained component represents the portion attributable to differences in coefficients, including unmeasured factors, changes in associations, or residual differences not captured by the observed variables. In this study, the explained component indicates the extent to which changes in measured characteristics contributed to the observed difference in OHIP-14 scores

## Discussion

Our study found that OHRQoL among Australian adults improved over 14 years, with mean OHIP-14 scores decreasing between survey waves. Despite this overall improvement, poorer OHRQoL continued to be associated with older age, female sex, lower education and household income, poorer self-rated general and oral health, and adverse oral health behaviours. Decomposition analysis showed that the observed improvement in OHRQoL was partly attributable to changes in socioeconomic composition and dental visiting patterns but was not fully explained by the measured factors.

Our findings showed a decrease in the mean OHIP-14 score from 7.3 in NSAOH-1 to 5.5 in NSAOH-2 among Australian adults, indicating an overall improvement in oral health-related quality of life (OHRQoL). This finding is consistent with previous Australian oral health reports showing improvements in some clinical and functional oral health indicators over time, including reductions in edentulism and non-functional dentition [21–24]. Improvements in dentition may have contributed to better perceived oral health by supporting chewing, eating, speaking, and social functioning. However, because this study used repeated cross-sectional survey data, these changes should be interpreted as population-level differences between survey waves rather than improvements observed within the same individuals over time. Although the improvement in OHRQoL coincided with national and state-level oral health initiatives and increased public awareness [22], this study did not directly assess policy exposure or system-level factors. Therefore, the findings should not be interpreted as evidence of policy impact.

The decomposition analysis showed that 41% of the difference in mean OHIP-14 scores was explained by measured population characteristics, while 59% remained unexplained. Household income and usual reason for dental visiting made the largest contributions to the explained component, followed by smoking status and education level. These findings suggest that changes in socioeconomic composition and dental visiting patterns partly contributed to the observed improvement in OHRQoL. However, the large unexplained component indicates that other factors may also have played a role.

The risk factors associated with OHRQoL identified in our study were consistent with those reported in international research. In terms of health-related factors, self-reported poor oral health was commonly associated with lower OHRQoL [23]. Among behavioural and lifestyle factors, tobacco smoking showed a strong association with periodontitis and tooth loss, both of which contribute to diminished OHRQoL [8]. Regarding socioeconomic and demographic factors, older age was linked to increased tooth loss, which can impair chewing and eating functions, thereby reducing OHRQoL [9]. Additionally, lower levels of education and income were associated with limited access to dental care and a higher burden of oral diseases, further contributing to poorer OHRQoL [15]. However, these associations should not be interpreted causally. Some covariates, such as dental visiting patterns or oral health status, may lie on causal pathways between socioeconomic position and OHRQoL.

## Strengths and limitations

A key strength of this study was the use of two large, nationally representative samples of Australian adults (with weighted data) to assess and compare OHRQoL over time, which helped to reduce random errors and minimise biased estimates. Nevertheless, several limitations should be acknowledged. The repeated cross-sectional design limits causal inference and prevents assessment of individual-level changes in OHRQoL over time. Differences in survey mode and data collection procedures may also have affected comparability between the two surveys. In addition, several variables were self-reported and may be subject to recall or reporting bias. Some potentially important determinants of OHRQoL were not measured or were not available in comparable form across both surveys, including detailed measures of dental service access and affordability, waiting times, culturally safe care, dental anxiety, satisfaction with dental professionals, and psychosocial factors such as stress, discrimination, and social support. These factors have been identified in previous reports and studies as important influences on dental attendance, oral disease experience, and oral health-related quality of life.

## Conclusion

OHRQoL among Australian adults improved over the 14 years, as shown by a decline in mean OHIP-14 scores between NSAOH-1 and NSAOH-2. However, poorer OHRQoL remained associated with older age, females, lower education and household income, poorer self-rated general and oral health, smoking, toothache, and problem-oriented dental visiting. Decomposition analysis showed that 41% of the difference in mean OHIP-14 scores was explained by measured population characteristics, particularly household income and usual reason for dental visiting. These findings suggest that improvements in OHRQoL were partly related to changes in socioeconomic composition and dental visiting patterns, while a substantial proportion remained unexplained. Overall, the findings indicate population-level improvement in perceived oral health outcomes, alongside persistent social and behavioural inequalities.

## Data Availability

The datasets generated and/or analysed during the current study are not publicly available due to privacy issues of the participants. Data is available from the corresponding author upon reasonable request.

## Abbreviations

CATI: Computer-assisted telephone interviews
CIs: Confidence intervals
NSAOH: National Survey of Adult Oral Health
OHIP-14: Oral Health Impact Profile 14
OHRQoL: Oral health-related quality of life
